# Effect of the Holiday Season on Weight Gain: A Narrative Review

**DOI:** 10.1155/2017/2085136

**Published:** 2017-07-04

**Authors:** Rolando G. Díaz-Zavala, María F. Castro-Cantú, Mauro E. Valencia, Gerardo Álvarez-Hernández, Michelle M. Haby, Julián Esparza-Romero

**Affiliations:** ^1^Department of Chemical and Biological Sciences, University of Sonora, Blvd. Luis Encinas y Rosales S/N, 83000 Hermosillo, SON, Mexico; ^2^Department of Medicine and Health Sciences, University of Sonora, Blvd. Luis Encinas y Rosales S/N, 83000 Hermosillo, SON, Mexico; ^3^Department of Public Nutrition and Health, Research Center for Food and Development (CIAD, A.C.), Road to Victoria km 0.6, 83000 Hermosillo, SON, Mexico

## Abstract

Several studies suggest that the holiday season, starting from the last week of November to the first or second week of January, could be critical to gaining weight. This study aims to review the literature to determine the effects of the holidays on body weight. In studies of adults, a significant weight gain was consistently observed during this period (0.4 to 0.9 kg, *p* < 0.05). The only study in college students found an effect on body fat but not on weight (0.1 kg, *p* = 0.71). The only study found in children did not show an effect on BMI percentile (−0.4%, *p* > 0.05) during this period. Among individuals with obesity who attempt to lose weight, an increase in weight was observed (0.3 to 0.9 kg, significant in some but not in all studies), as well as increase in weight in motivated self-monitoring people (0.4 to 0.6%, *p* < 0.001). Programs focused on self-monitoring during the holidays (phone calls and daily mailing) appeared to prevent weight gain, but information is limited. The holiday season seems to increase body weight in adults, even in participants seeking to lose weight and in motivated self-monitoring people, whereas in children, adolescents, and college students, very few studies were found to make accurate conclusions.

## 1. Introduction

Obesity is one of the most important health problems worldwide, making research on its causes a priority [1, 2].

Interest in the causes and prevention of obesity has increased due to the difficulty of treatment. Intensive lifestyle change programs, which are considered the gold standard, reduce weight by around 8–10% in one year [3], resulting in multiple benefits to health [4]. Nevertheless, weight loss over the long term (≈10 years) was 6% [3, 4], which means that the results are not completely maintained. In the case of pharmacotherapy, results for FDA-approved drugs ranged from 5.4 to 9.8% in one year [5], but long-term evaluations (>2 years) are lacking [6]. It has been assumed that the most efficient long-term treatment for obesity is bariatric surgery [7, 8], although this surgery is limited to more severe states (body mass index [BMI] > 40 or 35 with comorbidities) [8, 9]. In spite of how promising these procedures are, a recent systematic review of randomized controlled clinical trials has shown that there are few studies with this kind of design, that they have small sample sizes, that there is a possibility of adverse events following surgery, and that there is a lack of long-term evaluations (>2 years of follow-up) [10].

Further, once an individual achieves a weight loss, various neuroendocrine, metabolic, and behavioral mechanisms are set in motion to counteract the loss of weight [11, 12], in addition to the obstacles of having a healthy lifestyle in an obesogenic environment. Thus, it is necessary to consider prevention as the best strategy to fight obesity.

It is recommended that preventive interventions for obesity should aim to modify the environment and food system, as well as behavior-change communication through policy and regulation [13]. However, these actions do not consider specific periods in the year, such as the holiday season. This season, usually from the last week of November to the first or second week of January, could be a risky period for weight gain and obesity [14]. During this time, people from different countries attend religious and cultural celebrations such as Christmas and New Year, among other social gatherings. Special meals and high-energy dense foods are prepared in this period: desserts, candies, sugary drinks, or alcohol. There is also evidence of a decrease in physical activity at this time [15, 16].

Sixteen years ago an article published by Yanovski and colleagues showed that a nonrepresentative sample of 195 adults gained an average of 0.37 kg during the holiday season [14]. The researchers found that weight gain during this period is not subsequently lost in a year and represents more than 50% of the weight gained throughout the year [14]. Additionally, other studies have suggested that this period could be critical in weight gain, mainly in adults. This study aimed to review the evidence on the effect of the holidays on body weight.

## 2. Methods

This is a narrative (not systematic) review of the topic that highlights some of the main findings in the area and discusses potential areas for further research. A search was conducted in PubMed and other databases, using the keywords “holiday/holidays" and “weight gain/obesity.” The search was limited to studies published in English and Spanish without specifying limits on dates, study design, or the age of the participants.

The exposure of interest is defined as the holiday season, typically starting from the last week of November to the first or second week of January. To be selected, studies had to include at least one measurement of weight or BMI before and after the holiday season.

Once the articles were identified, some data were extracted and checked. Information extracted from the selected studies included author, year, and country of study; number of participants; date of measurements; sample selection, retention of subjects, and masking of participants; and change in weight or BMI. We did not undertake a formal quality assessment but do consider the important methodological characteristics when interpreting the results. Results are reported narratively. Finally, we reviewed some relevant articles related to the effect of other festive periods on body weight and the possible causes of weight gain during these periods for discussion purposes only.

## 3. Results

Fifteen publications were included [14, 17–30], six were in the adult population [14, 18, 21–24], six were in adults seeking to lose weight [17, 19, 25, 28–30], one in motivated self-monitoring people [20], one in college students [27], and one in children [26]. All of the studies were conducted in the northern hemisphere. A summary of the results of the effects of the holiday season on body weight in adults is shown in Table 1; the results of the studies with adults who were seeking to lose weight or motivated self-monitoring people are shown in Table 2, while Table 3 shows the results in college students and children. No studies were found in adolescents. The following are the results of the studies described chronologically by population group.

### 3.1. Studies in Adults

In 1985, Rees et al. reported the effects of the holidays on body weight and biochemical parameters in a small group of healthy subjects (*n* = 22) and another with type 2 diabetes (*n* = 13) [21]. Volunteers were measured five times (December 10, 22, and 29 and January 5 and 22). From baseline, all subjects significantly gained an average of 0.8 kg. Subjects with diabetes gained 0.7 kg despite being on a diet or medication, while healthy participants gained 0.9 kg. There was a healthy woman who gained 4.3 kg. The highest weight gain was observed from December 22 to 29 and was maintained until the last measurement. A significant increase in glycated hemoglobin (+0.7% healthy group, +1.1% diabetes group), total cholesterol, low density lipoprotein, insulin, and triglycerides in the study period was also observed, suggesting an increase in dietary energy intake. It is worth mentioning that participants were laboratory technicians, paramedics, nurses, doctors, or friends of those mentioned. Subjects with diabetes had an exemplary record in their glycemic control [21].

In 1999, Reid and Hackett assessed the effect of the holidays on body weight in 26 healthy volunteers between 17 and 59 years of age with an average BMI of 20.5 kg/m^2^. The initial measurement was recorded from December 18th to 21th and the final was around January 4th. A significant average increase of 0.9 kg was observed in this short period (around 15 days) [22].

In 2000, Yanovski et al. evaluated 195 adults before and after the holiday season (last week of November to mid-January) and participants showed an increase in their weight of 0.37 kg during this short period of 6 weeks [14]. For the 165 subjects who returned for the one-year measurement, it was found that the weight gained during the holiday period was not subsequently lost. The subjects increased 0.62 kg over the course of a year, so the increase in the holiday season (0.32 kg in this group) represented about 50% of the weight gained in all the year [14]. However, a convenience sample was used; thus it is not representative of the general population.

In 2012, Wagner et al. studied the effects of the holidays on weight and body composition in 13 men and 21 women aged between 23 and 61 years [24]. The initial measurement was taken in mid-November, while the second was taken in early January. Although the participants perceived that they had gained weight, no significant changes were observed in weight before and after the holiday period (74.0 kg versus 73.9 kg, difference −0.1 kg, *p* = 0.87) nor in body fat (25.4% versus 25.4%, difference 0.0%, *p* = 0.97) [24].

Also in 2012, a secondary analysis of a study originally conducted to determine the error rate of dietary self-report questionnaires against total energy expenditure measured with doubly labeled water was published by Cook and colleagues [18]. In this study, 443 men and women aged 40 to 69 years and with an average BMI of 27 kg/m^2^ for women and 28 kg/m^2^ for men were measured. Initial measurements were conducted in the period from mid-September to mid-October and the final measurement from mid-January to early March. It was observed that participants significantly increased their weight (average 0.8 kg) during the study period [18].

In 2013, Stevenson et al. conducted a study with 148 participants to assess if exercise performed regularly during the Christmas period had an effect on body weight [23]. The initial assessment was made in mid-November and the final assessment in mid-January. Measurements included body weight, fat percentage, blood pressure, and self-reported exercise. Participants gained 0.78 kg (*p* < 0.001) and increased their fat percentage by 0.5% (*p* = 0.007). It should be noted that obese subjects showed a greater increase in fat percentage compared to normal weight subjects (1.6% versus 0.2%, resp., *p* < 0.05), but no difference was noted in body weight compared to normal weight subjects. Regular self-reported exercise was not shown to be a protective factor against weight gain during this period and was not a significant predictor of change in body weight or body fat percentage [23].

### 3.2. Studies in Adults with Obesity Who Attempt to Lose Weight or Motivated Self-Monitoring People

The negative effect of the holidays on body weight also seems to be observed in patients seeking to lose weight.

Andersson and Rössner, in 1992 conducted a study that included 46 patients with obesity participating in a maintenance therapy program at the obesity unit of a hospital in Sweden. These patients self-reported their weight weekly in individual charts where the information of the period of the holidays was obtained (before Christmas to mid-January, three-week period). In addition, a control group of subjects from the hospital staff were included. They were invited to record their weight during the same period. Patients with obesity had a nonsignificant increase of 0.6 ± 2.4 kg, whereas controls had an increase of 0.4 ± 0.8 kg (*p* < 0.001) in the 3-week period [28].

In 1999, Boutelle et al. evaluated an intervention to increase consistency of self-monitoring via mailings and phone calls during the period of the holiday season [17]. Fifty-seven subjects who participated in a long-term cognitive behavioral program that included self-monitoring were randomly assigned to increase consistency of self-monitoring during the holiday season or to a control group. Measurements were taken from the beginning of December until the end of January. The intervention group had a greater self-monitoring and achieved a better control of body weight compared to the control group (−0.9 kg versus 0.9 kg, *p* = 0.007), during the 8 weeks of intervention [17]. A previous study by Baker and Kirschenbaum showed that individuals participating (50 weeks) in a behavioral intervention had a mean weight loss of 12 kg but increased 500% more weight per week during holidays in comparison with nonholiday weeks. Also, only individuals in the high quartile of self-monitoring were able to successfully lose weight during this period [31].

In 2007, Watras et al. reported the weight change during the holiday season of individuals who participated in a double-blind, randomized placebo-controlled study that evaluated the effect of conjugated linoleic acid on fat loss for 6 months. The investigators observed that individuals in the conjugated linoleic acid group reduced their weight by 0.1 kg, whereas those in the placebo group increased their weight by 0.6 kg during this season (*p* < 0.05) [25].

In 2007, Squires described the experience from the Lean Plate Challenge [30]. This program provided support for participants to maintain their weight from Thanksgiving to New Year. The subjects recorded their weight and received information about the challenge and goals on diet and physical activity. They also received support at their workplace (cafeterias agreed to make reduced calorie meals during the period of the challenge) and other incentives when they finished the challenge (a t-shirt and one ticket to participate in a drawing to win a mountain bike). The number of participants that were able to finish the challenge was 217 (from 290) and a little more than 60% achieved the maintenance goal, with an average loss of 1.0 kg (significance not reported) [30].

In 2008, Phelan and colleagues evaluated the effect of the holiday period in a group of successful weight losers from the National Weight Control Registry [29]. This group had a mean weight loss of 34.9 kg and had maintained >13 kg for almost 6 years. In addition, researchers evaluated a group of subjects with normal weight (BMI of 18.5 to 24.9 kg/m^2^) with no history of obesity. Both groups increased body weight in the holiday period (0.7 kg versus 0.2 kg) with no significant differences between them. However, more successful weight losers gained more than 1 kg, compared to the normal weight group (39% versus 17%) during the holiday period [29].

A study in Spain, by García et al., showed that 215 patients participating in a clinical treatment for obesity gained 0.29 kg weight at Christmas and New Year's Eve (25 December to 5 January) even when they were prescribed to eat a hypocaloric diet (restriction of 500–600 kcal/day) [19].

In 2016, Helander et al. reported weight changes throughout the year in 2924 Withings weight scale users from 3 developed countries (Unites States [US], Germany, and Japan); participants also created an online account to monitor their weight. The users' weight increased significantly between 10 days before Christmas and 10 days after Christmas (US 0.4% increase in weight, *p* < 0.001; Japan 0.5%, *p* = 0.005; and Germany 0.6%, *p* < 0.001). A significant increase was also observed in weight on other national holidays compared to preholiday weight: Japan during Golden Week (0.3%, *p* < 0.001), Germany over Easter holidays (0.2%, *p* < 0.001), and the US during Thanksgiving (0.2%, *p* < 0.001). Half of the weight gained during the Christmas holidays was quickly lost and participants in the three countries lost weight during the year of study. The individuals reported an average of 147.5 to 181.6 weigh-ins over the year, suggesting that these groups represent actively self-monitoring people, rather than the general population. Despite being a group of highly motivated individuals (as seen by the number of weigh-ins and amount of weight loss), the effect of the holiday periods on body weight was marked [20].

### 3.3. Studies in College Students

In 2006, Hull et al. studied a convenience sample of 94 young people attending college with an average age of 23 years (Table 3). A weight gain of 0.5 kg, 13 days after the Thanksgiving day period (last week of November) was observed [32]. However, the weight gained during this period was quickly lost at the next measure from January 9 to 20. No difference was found between the initial weight and final weight in January (71.3 kg versus 71.2 kg, difference −0.1 kg, *p* = 0.71). However, there was a significant increase in the percentage of body fat measured by dual X-ray absorptiometry (25.9 versus 27.0, *p* < 0.05) [27].

### 3.4. Studies in Children

In 2010, Branscum et al. studied the effect of the holidays on obesity parameters in 90 children from a public elementary school in the United States. The study included two measurements: one in early December (before the holiday period) and one in early to mid-January (at the return to classes). During this time the children gained 0.56 kg and increased their BMI by 0.28 kg/m^2^ but an increase in height of 0.82 cm was also observed. Thus, on average, there was no change in BMI percentile (−0.4%, *p* > 0.05). It was noted that children with overweight and obesity increased their body mass index percentile and BMI and weigh more than children with normal weight [26].

## 4. Discussion

Adult studies consistently showed an increase in body weight during the holiday period, with the exception of one study [24] with very few participants in which there was no increase (−0.1 kg). One study found an increase of 0.37 kg [14], and another four studies showed an increase between 0.58 to 0.9 kg [18, 21–23]. Results are similar to those reported in a review of the literature by Schoeller in 2014 that found an increase in weight of around 0.5 kg [33]. Our work is different from the Schoeller review because we have also included studies of populations seeking to lose weight and from a wider variety of countries. Additionally, we discuss studies that assessed the effect of other festive periods on body weight (see next section). In contrast to adult populations, no increase in weight was found in the college population (one study) but there was a significant increase in body fat [27]. In the case of children, one study showed no effect of the holiday period on BMI percentile (−0.4%, *p* > 0.05) [26]. Individuals seeking to lose weight showed a mean increase from 0.3 kg to 0.9 kg (significant in some but not in all studies). Finally, motivated self-monitoring people from three countries also significantly increased their body weight. It should also be noted that, in general, participants who are overweight or obese gain more weight than those of normal weight [14, 23, 26, 27, 34], which should be considered in the planning of preventive measures.

The reported results derive mainly from the US, England, and other developed countries and may not be generalizable to other countries due to different factors that might influence diet and physical activity in the holiday period. For example, in México workers receive a higher income (at least 15 to 50 days' salary) during December, which would allow more food purchases. There are also differences in religious and cultural festivities, and environmental, socioeconomic, and climatic factors can be markedly different. However, it is worth noting that studies that included samples from other countries outside of the US and England (i.e., Germany, Japan, Sweden, and Spain) also showed that participants gained weight in the holiday season [19, 20, 28].

### 4.1. Effect of Other Festive Periods on Body Weight

Recently, Cooper and Tokar showed that other holiday periods (from one to three weeks away from the place of residence) between March and August also increased body weight and this was maintained 6 weeks later [35]. Also, the length of vacation had a direct relationship with increasing weight [35]. These results were observed even though individuals reported increased total physical activity during the holiday period, suggesting that the cause is the increased intake of dietary energy [35]. Hourdakis et al., meanwhile, assessed the effect of the Greek Orthodox Easter holidays in a sample of 138 college students [34]. The results showed an increase of 1.5 to 1.7 kg (*p* < 0.001) in men and women, respectively [34]. Payab et al. ascertained the effects of the Persian New Year Festival “Norouz,” which takes place at the beginning of spring [36]. Sixty-six subjects, aged 21–68 years participated in anthropometric measurements in mid-March and April (approximately 45 days apart). A significant increase in weight of 0.58 kg (*p* < 0.001) was observed [36].

In relation to studies in children, Cristi-Montero et al. evaluated the effect of two vacation periods (national and winter) on some parameters of obesity in Chilean schoolchildren, age 10 to 14 years, with medium socioeconomic status [37]. For the national holidays (9 days of September) 147 schoolchildren participated and for the winter holidays (16 days of July) 216 schoolchildren participated. Weight (kg) and fat (percentage, estimated from skinfolds) gained during the national holiday were 0.6 kg, *p* < 0.001, and 0.51%, *p* = 0.0013, respectively, with no change in waist circumference. Concomitantly, a reduction in self-reported physical activity and an increase in self-reported sleeping time were observed. In the winter holidays schoolchildren gained weight (0.51 kg, *p* < 0.001), fat (0.51%, *p* < 0.001), and increased waist circumference (0.65 cm, *p* < 0.001), simultaneous with an increase in the self-reporting of their sleeping time, with no change in physical activity reported [37]. In this study the authors did not measure or account for any change in height during the study period that could be related to weight change. However, the authors argued that they had carried out a pilot study to estimate the weight change during a “normal” week in a random sample of 216 students of the same age, finding that there was no change in body weight (0.04 kg, *p* = 0.12) [37].

Finally, Baranowski et al. conducted a narrative review of differences in weight gain (BMI *Z* score, obesity, or other parameters) between the school year and summer holidays in children. The common pattern in six longitudinal studies was that children with overweight and obesity had accelerated weight gain during the summer, while normal weight children gained less or did not gain weight [38]. Four intervention studies were included (three fitness interventions [39–41] and one behavioral weight loss program [42]) where they found the benefits from fitness parameters were lost during summer. Franckle et al., in their systematic review of the literature, also found accelerated weight gain is observed during summer, especially in Afro-American, Hispanics, and overweight children and adolescents [43]. Additionally, Zinkel et al. did not find any significant change in total energy expenditure (adjusted for fat-free mass) and physical activity level over summer versus in school months among children from the United States using the doubly labeled water method. This suggests that the reason for the weight gain during summer is not necessarily related to differences in energy expenditure [44].

### 4.2. Possible Causes of Weight Gain during the Holiday Period

The causes that could explain a weight gain during the holiday period are diverse. Typically, at this time people have a more carefree lifestyle, special meals at parties are prepared, and social gatherings can be more frequent, which exposes them to situations that increase food intake: greater variety of foods [45], high-energy dense foods [46], bigger portion sizes (i.e., buffets) [47], and eating in company (i.e., social reunions) [48], plus less physical activity during winter [15, 16]. However, there is a lack of studies that have elucidated the mediators of the weight gain during these periods.

There are mechanisms that regulate weight at an established point [49]. However, this mechanism appears to be asymmetrical, more oriented to the prevention of weight loss (body fat) rather than to prevention of weight gain [49, 50]. The Westernized “Cafeteria diet” that includes palatable and high calorie foods may also be common during holidays. In experimental animal model studies, this diet can cause loss of weight regulation, contributing to hyperphagia and obesity [49, 51, 52]. This palatable diet can lead to high intakes of food by disrupting diverse signaling pathways related to food control (including the activation of the reward system) [52].

### 4.3. Effects of Weight Gain, Weight Loss, and Weight Regain (Yoyo Effect)

The deleterious effect of weight gain and obesity on health is highly documented. However, the effect of losing and regaining weight or gaining and reducing weight is a different case. A recent study showed that fluctuations in body weight are associated with a higher rate of cardiovascular events and mortality in individuals with coronary artery disease [53]. However, the point that the yoyo effect is harmful to health remains debatable [54–56]. It is necessary to make a distinction between variations in body weight for various reasons and fluctuations in body weight due to voluntary healthy lifestyle intervention. When an individual regains some of his weight after participating in a lifestyle intervention program (healthy diet, regular physical activity, and behavior therapy), the evidence shows that this body weight fluctuation is associated with positive effects on health. The Diabetes Prevention Program study showed that at one year after randomization, participants belonging to the lifestyle intervention group lost 7 kg; nevertheless at year 5 the weight loss was only 2 kg, and there were no differences versus the control group at year 10 after randomization [57]. However, the cumulative incidence of type 2 diabetes was reduced by 34% in the lifestyle intervention versus the control group [57]. It makes sense that when individuals with obesity improve their weight, fitness, body composition, blood lipids, blood sugar, and blood pressure, lower inflammation, and reduce sleep apnea, liver fat, and urinary incontinence as well as improving sexual dysfunction and other markers of mental health (depression and quality of life) [4] even for a limited or an extended time, these benefits could impact positively their health instead of harming it.

## 5. Limitations of the Studies and Recommendations

Selection bias is among the limitations of the studies reviewed, for instance, in using nonrandom and nonrepresentative samples. People interested in their weight and health could be more likely to be involved in these studies, leading to more conservative results. However, it has been observed that even health practitioners and patients with controlled diabetes, highly motivated individuals and participants under treatment for obesity also gain weight during the holiday season (Table 2). Small sample size is another potential source of bias, with most studies having a sample of less than 200 participants. Most of the participants knew that the main outcome of the study was to evaluate the effect of holidays on body weight, likely making them more conscious about their weight. Although studies suggest weight might be gained during holidays, only two studies have followed up the participants to see if this weight is maintained or subsequently lost [14, 20]. In the Yanovski study the weight gain was maintained afterwards [14], while the motivated self-monitoring group of Withings weight scale users lost the weight gained during the holiday season in the follow-up period [20]. Thus, more studies are required with follow-up of the participants beyond the holiday season to evaluate if the weight gain is maintained or lost.

## 6. Conclusion

Holidays seem to increase body weight in adults. However, to be able to generalize the results, studies with representative population samples are needed. In children, adolescents, and young adults (college students) there are few published studies from which to draw conclusions. Participants seeking to lose weight appeared to increase weight although this was not consistently significant and motivated self-monitoring people also appeared to increase weight. These results must be considered for registered dietitian nutritionists, other health providers, and policy makers to prevent weight gain in their patients and communities during this critical period. Studies from other countries are required, given that the vast majority of the studies were conducted in the United States and United Kingdom. We suggest more studies are needed that include representative samples of the general population in different age groups that evaluate change in obesity parameters and that consider the methodological limitations mentioned in this review.

## Figures and Tables

**Table 1:** Studies evaluating the effect of the holiday period on body weight in adults.

Author, year, and country	Number of participants, age, sex, BMI, and prevalence of overweight/obesity	Date of measurements^a^	Sample, masking of participants, retention^b^	Mean weight change ± SD	*p* value
Rees et al. 1985 United Kingdom	22 healthy adults and 13 adults with T2DM (age, sex, BMI, and prevalence not reported)	Measurements: December 10, 22 and 29. January 5 and 26.	Convenience sample. No. Retention 76%.	Healthy = 0.9 ± 1.0 kg T2DM = 0.7 ± 0.6 kg	*p* < 0.001

Reid and Hackett 1999 United Kingdom	26 adults 17–59 years Men = 42% Women = 58% BMI = 20.5 ± 3.28 kg/m^2^ Low weight = 23% Normal weight = 65% Overweight = 12%	Initial measurement: December 18 to 21. Final measurement: January 4.	Convenience sample. No. Retention 96%.	0.93 ± 1.2 kg	*p* < 0.05

Yanovski et al. 2000 United States	195 adults 19–82 years Men = 49% Women = 51% BMI 25.9 ± 4.8 kg/m^2^ Normal weight = 52% Overweight = 27% Obese = 21%	Initial measurement: in mid-November. Final measurement: in early January.	Convenience sample. Yes. Retention 98%.	0.37 ± 1.5 kg	*p* < 0.001

Wagner et al. 2012 United States	34 adults 23–61 years Men = 41% Women = 59% BMI = 25.3 ± 5.3 kg/m^2^ Normal weight = 65% Overweight = 35%	Initial measurement: November 24-25. Final measurement: January 5-6.	Convenience sample. No. Retention 92%.	−0.1 ± 1.48 kg	*p* = 0.87

Cook et al.^c^ 2012 United States	443 adults 40–69 years Men = 55% Women = 45% BMI man = 28 ± 4 kg/m^2^ BMI woman = 27 ± 6 kg/m^2^ Normal weight = 30% Overweight = 42% Obese = 28%	Initial measurement: mid-September–mid-October. Final measurement: mid-January–early March.	Convenience sample. Yes. Secondary analysis. Retention 98%.	Man = 0.9 ± 1.4 kg Woman = 0.6 ± 1.3 kg	*p* < 0.05

Stevenson et al. 2013 United States	148 adults 18–65 years Men = 32% Women = 68% BMI = 25.1 ± 0.5 kg/m^2^ Normal weight = 57% Overweight = 26% Obese = 17%	Initial measurement: mid-November. Final measurement: mid-January.	Convenience sample. Yes. Retention 70%.	0.78 ± 0.1 kg	*p* < 0.001

^a^Weight of participants in all studies was measured as part of the study. ^b^Masking: the participants did not know that the study objective was to evaluate the effect of holidays on obesity parameters. ^c^The winter holiday season period was not specifically measured; however, the evaluation covered the month of December; BMI: body mass index. T2DM: type 2 diabetes mellitus.

**Table 2:** Studies evaluating the effect of the holiday period on body weight in adults attempting to lose weight or motivated self-monitoring people.

Author, year, and country	Number of participants, age, sex, BMI, and prevalence of overweight/obesity	Date of measurements^a^	Sample, masking of participants, retention^b^	Mean weight change ± SD	*p* value^c^
Andersson and Rössner 1992 Sweden	46 adults 20–70 years Men = 28% Women = 72% BMI = 36.8 ± 6.6 kg/m^2^ Obese = 100% 76 adults 20–70 years Men = 28% Women = 72% BMI = 22.9 ± 2.6 kg/m^2^ Obesity not reported	Initial measurement: before Christmas. Final measurement: after January 6th. (3 weeks between data collected).	Subjects participating in a maintenance program for obesity. Masking only in this group. Subjects from the hospital staff who participated as control group (not attempting to lose weight). Yes. Retention not reported.	0.6 ± 2.4 kg 0.4 ± 0.8 kg	NS *p* < 0.001

Boutelle et al. 1999 United States	57 adults 44.5 ± 10.0 years Men = 28% Women = 72% BMI = 35.5 ± 7.3 kg/m^2^ Obese = 100% (initial BMI 35.5 ± 7.3 kg/m^2^ and a mean weight loss of 15.1 ± 12.4 kg)	Specific dates not reported, but describe changes during 8 weeks of holiday season (early December to last days of January).	Subjects participating in long-term cognitive-behavioral treatment program, assigned to receive an intervention for improving consistency of self-monitoring. Subjects participating in long-term cognitive-behavioral treatment program without receiving the intervention for improving consistency of self-monitoring. No. Retention 88%.	−0.9 ± 2.45 kg 0.9 ± 3.49 kg	*p* = 0.007 (between groups comparisons)

Watras et al. 2007 United States	22 adults 18–44 years Men = 23% Women = 77% BMI = 27.6 ± 1.8 kg/m^2^ Overweight = 100% 18 adults 18–44 years Men = 17% Women = 83% BMI = 28.0 ± 2.2 kg/m^2^ Overweight = 100%	Specific dates of measurements not described, but reported the increase in kg/month during holiday season (November and December).	Subjects who participated in a randomized trial evaluating conjugated linoleic acid for fat loss Subjects from the placebo control group. No. Retention 83%.	−0.1 kg^d^ 0.6 kg^d^	*p* = 0.01 (between groups comparisons)

Squires 2007 United States	217 adults Age, sex, BMI, and prevalence of overweight/obesity not reported	Initial measurement: at the beginning of the challenge. Final measurement: at the end of the challenge. (data collected between Thanksgiving and New Year).	Subjects participating in a weight maintenance challenge. No. Retention 75%.	−1.0 kg^d^	NR

Phelan et al. 2008 United States	167 adults 47.5 ± 11.5 years Men = 25% Women = 75% BMI = 25.5 ± 6.4 kg/m^2^ Obese = 100%, (maximum BMI = 37.4 ± 9.4 kg/m^2^ and a mean weight loss of 34.9 ± 16.5 kg) 90 adults 45.5 ± 13.9 years Men = 25% Women = 75% BMI = 21.8 ± 1.9 kg/m^2^ Normal weight = 100%	Initial measurement: early November. After holiday measurement: early January.	Successful weight losers from the National Weight Control Registry. Normal weight individuals not attempting to lose weight. No. Retention 94% and 89%.	0.7 ± 1.8 kg 0.2 ± 1.0 kg	NS NS

García et al. 2013. Spain	215 adults 14–70 years Men = 37% Women = 63% BMI = 28.0 ± 2.22 kg/m^2^ Overweight or obese = 100%	Initial measurement: a couple of days before December 25th. Final measurement: a couple of days after January 5th. (2 weeks between measurements).	Individuals receiving treatment for obesity No. From 258 patients, 215 were measured on both occasions.	0.29 ± 1.33 kg	*p* < 0.05

Helander et al. 2016. United States, Germany and Japan	United States: 1781 adults Age = 42.2 years Men = 66% Women = 34% BMI = 27 ± 5.2 kg/m^2^ Obese = 24% Germany: 760 adults Age = 42.9 years Men = 66% Women = 34% BMI = 26.6 ± 4.9 kg/m^2^ Obese = 19% Japan: 383 adults Age = 41.6 years Men = 74% Women = 26% BMI = 24.7 ± 4.7 kg/m^2^ Obese = 11%	Initial measurement: ten days before Christmas. Final measurement: ten days after Christmas.	From a random sample of 10,000 Withings weight scale users, 2924 subjects were analyzed after the exclusion criteria were applied. Yes.	United States: 0.4% kg^d,e^ Germany: 0.6% kg^d,e^ Japan: 0.5% kg^d,e^	*p* < 0.001 *p* < 0.001 *p* = 0.005

^a^Weight of participants in all studies was measured as part of the study, except for Andersson and Rössner 1992 (self-report in individual charts), Phelan et al. 2008 (self-report via questionnaire), and Helander et al. 2016 (scale saves the weight of the individual and sends it to network servers). ^b^Masking: the participants did not know that the study objective was to evaluate the effect of holidays on obesity parameters; ^c^*p* value derived from comparisons before and after analysis, except when specified; ^d^SD not reported. ^e^Weight reported by percent of weight, because authors of the study did not report kilograms; BMI: body mass index. NS: not significant. NR: not reported.

**Table 3:** Studies evaluating the effect of the holiday period on body weight in children and college students.

Author, year, and country	Number of participants, mean age, sex, BMI, and prevalence of overweight/obesity	Date of measurements^a^	Sample, masking of participants, retention^b^	Change in obesity parameter	*p* value
Branscum et al. 2010 United States	88 children (average age 9.1 years) Boys = 53% Girls = 47% BMI percentile = 73.9 ± 26.0 Normal weight = 51% Overweight/obesity = 49%	Initial measurement: in early December. Final measurement: in mid-January.	Convenience sample. No. Retention 98%.	BMI percentile = reduction 0.65	*p* > 0.05

Hull et al. 2006 United States	82 university students (18–40 years) Men = 45% Women = 55% BMI = 23.9 ± 4 kg/m^2^ Normal weight = 66% Overweight/obesity = 34%	Initial measurement: November 14 to 22. Final measurement: January 9 to 21.	Convenience sample. No. Retention 82%.	Weight = −0.1 kg	*p* = 0.71

^a^Weight of participants in all studies was measured as part of the study. ^b^Masking: the participants did not know that the study objective was to evaluate the effect of holidays on obesity parameters; BMI: body mass index.

## References

[1] Scully T. (2014). Public health: society at large. *Nature*.

[2] Frühbeck G., Toplak H., Woodward E., Yumuk V., Maislos M., Oppert J.-M. (2013). Obesity: the gateway to ill health - an EASO position statement on a rising public health, clinical and scientific challenge in Europe. *Obesity Facts*.

[3] The Look AHEAD Research Group (2013). Cardiovascular effects of intensive lifestyle intervention in type 2 diabetes. *New England Journal of Medicine*.

[4] Pi-Sunyer X. (2014). The look AHEAD trial: a review and discussion of its outcomes. *Current Nutrition Reports*.

[5] Bray G. A., Frühbeck G., Ryan D. H., Wilding J. P. H. (2016). Management of obesity. *The Lancet*.

[6] Yanovski S. Z., Yanovski J. A. (2014). Long-term drug treatment for obesity: a systematic and clinical review. *Journal of the American Medical Association*.

[7] Ochner C. N., Tsai A. G., Kushner R. F., Wadden T. A. (2015). Treating obesity seriously: When recommendations for lifestyle change confront biological adaptations. *The Lancet Diabetes and Endocrinology*.

[8] Frühbeck G. (2015). Bariatric and metabolic surgery: a shift in eligibility and success criteria. *Nature Reviews Endocrinology*.

[9] Fried M., Yumuk V., Oppert J. M. (2014). Interdisciplinary European guidelines on metabolic and bariatric surgery. *Obesity Surgery*.

[10] Gloy V. L., Briel M., Bhatt D. L. (2013). Bariatric surgery versus non-surgical treatment for obesity: a systematic review and meta-analysis of randomised controlled trials. *British Medical Journal*.

[11] Greenway F. L. (2015). Physiological adaptations to weight loss and factors favouring weight regain. *International Journal of Obesity*.

[12] Sumithran P., Proietto J. (2013). The defence of body weight: a physiological basis: for weight regain after weight loss. *Clinical Science*.

[13] Roberto C. A., Swinburn B., Hawkes C. (2015). Patchy progress on obesity prevention: emerging examples, entrenched barriers, and new thinking. *The Lancet*.

[14] Yanovski J. A., Yanovski S. Z., Sovik K. N., Nguyen T. T., O'Neil P. M., Sebring N. G. (2000). A prospective study of holiday weight gain. *The New England Journal of Medicine*.

[15] Ma Y., Olendzki B. C., Li W. (2006). Seasonal variation in food intake, physical activity, and body weight in a predominantly overweight population. *European Journal of Clinical Nutrition*.

[16] Rich C., Griffiths L. J., Dezateux C. (2012). Seasonal variation in accelerometer-determined sedentary behaviour and physical activity in children: a review. *International Journal of Behavioral Nutrition and Physical Activity*.

[17] Boutelle K. N., Baker R. C., Kirschenbaum D. S., Mitchell M. E. (1999). How can obese weight controllers minimize weight gain during the high risk holiday season? By self-monitoring very consistently. *Health Psychology*.

[18] Cook C. M., Subar A. F., Troiano R. P., Schoeller D. A. (2012). Relation between holiday weight gain and total energy expenditure among 40- to 69-y-old men and women (OPEN study). *The American Journal of Clinical Nutrition*.

[19] García C. G., Berná A., Sebastià N., Soriano J. M. (2013). Prospective study on the effect of the influence of holiday periods in the weight during a low-calory dietetic treatment. *Nutrición hospitalaria*.

[20] Boyce T. G. (2016). Weight gain over the holidays in three countries. *New England Journal of Medicine*.

[21] Rees S. G., Holman R. R., Turner R. C. (1985). The Christmas feast. *British Medical Journal (Clinical research ed.)*.

[22] Reid R., Hackett A. F. (1999). Changes in nutritional status in adults over Christmas 1998. *Journal of Human Nutrition and Dietetics*.

[23] Stevenson J. L., Krishnan S., Stoner M. A., Goktas Z., Cooper J. A. (2013). Effects of exercise during the holiday season on changes in body weight, body composition and blood pressure. *European Journal of Clinical Nutrition*.

[24] Wagner D. R., Larson J. N., Wengreen H. (2012). Weight and body composition change over a six-week holiday period. *Eating and Weight Disorders - Studies on Anorexia, Bulimia and Obesity*.

[25] Watras A. C., Buchholz A. C., Close R. N., Zhang Z., Schoeller D. A. (2007). The role of conjugated linoleic acid in reducing body fat and preventing holiday weight gain. *International Journal of Obesity*.

[26] Branscum P. W., Kaya G., Succop P., Sharma M. (2010). An evaluation of holiday weight gain among elementary-aged children. *Journal of Clinical Medicine Research*.

[27] Hull H. R., Hester C. N., Fields D. A. (2006). The effect of the holiday season on body weight and composition in college students. *Nutrition Metabolism*.

[28] Andersson I., Rössner S. (1992). The Christmas factor in obesity therapy. *International Journal of Obesity*.

[29] Phelan S., Wing R. R., Raynor H. A., Dibello J., Nedeau K., Peng W. (2008). Holiday weight management by successful weight losers and normal weight individuals. *Journal of Consulting and Clinical Psychology*.

[30] Squires S. (2007). Controlling holiday weight gain: lessons from the lean plate club. *Journal of Nutrition Education and Behavior*.

[31] Baker R. C., Kirschenbaum D. S. (1998). Weight control during the holidays: Highly consistent self-monitoring as a potentially useful coping mechanism. *Health Psychology*.

[32] Hull H. R., Radley D., Dinger M. K., Fields D. A. (2006). The effect of the thanksgiving holiday on weight gain. *Nutrition Journal*.

[33] Schoeller D. A. (2014). The effect of holiday weight gain on body weight. *Physiology & behavior*.

[34] Hourdakis M., Papandreou D., Malindretos P. (2010). Effect of Greek orthodox easter holidays on body weight gain. *Nutrition and Food Science*.

[35] Cooper J. A., Tokar T. (2016). A prospective study on vacation weight gain in adults. *Physiology and Behavior*.

[36] Payab M., Hasani-Ranjbar S., Zahedi H. (2015). The effect of Norouz holiday on anthropometric measures and body composition. *Journal of Diabetes and Metabolic Disorders*.

[37] Cristi-Montero C., Bresciani G., Alvarez A. (2014). Critical periods in the variation in body composition in school children. *Nutricion Hospitalaria*.

[38] Baranowski T., O'Connor T., Johnston C. (2014). School year versus summer differences in child weight gain: a narrative review. *Childhood Obesity*.

[39] Carrel A. L., Clark R. R., Peterson S., Eickhoff J., Allen D. B. (2007). School-based fitness changes are lost during the summer vacation. *Archives of Pediatrics and Adolescent Medicine*.

[40] Gutin B., Yin Z., Johnson M., Barbeau P. (2008). Preliminary findings of the effect of a 3-year after-school physical activity intervention on fitness and body fat: The Medical College of Georgia Fitkid Project. *International Journal of Pediatric Obesity*.

[41] Yin Z., Moore J. B., Johnson M. H., Vernon M. M., Gutin B. (2012). The impact of a 3-year after-school obesity prevention program in elementary school children. *Childhood Obesity*.

[42] Gillis L., McDowell M., Bar-Or O. (2005). Relationship between summer vacation weight gain and lack of success in a pediatric weight control program. *Eating Behaviors*.

[43] Franckle R., Adler R., Davison K. (2014). Accelerated weight gain among children during summer versus school year and related racial/ethnic disparities: a systematic review. *Preventing Chronic Disease*.

[44] Zinkel S. R. J., Moe M., Stern E. A. (2013). Comparison of total energy expenditure between school and summer months. *Pediatric Obesity*.

[45] Rolls B. J., Rowe E. A., Rolls E. T., Kingston B., Megson A., Gunary R. (1981). Variety in a meal enhances food intake in man. *Physiology and Behavior*.

[46] Sampey B. P., Vanhoose A. M., Winfield H. M. (2011). Cafeteria diet is a robust model of human metabolic syndrome with liver and adipose inflammation: comparison to high-fat diet. *Obesity*.

[47] Rolls B. J., Morris E. L., Roe L. S. (2002). Portion size of food affects energy intake in normal-weight and overweight men and women. *American Journal of Clinical Nutrition*.

[48] de Castro J. M., Brewer E. M. (1992). The amount eaten in meals by humans is a power function of the number of people present. *Physiology and Behavior*.

[49] Müller M. J., Bosy-Westphal A., Heymsfield S. B. (2010). Is there evidence for a set point that regulates human body weight?. *F1000 Medicine Reports*.

[50] Rosenbaum M., Leibel R. L. (2014). Role of leptin in energy homeostasis in humans. *Journal of Endocrinology*.

[51] Rothwell N. J. (1997). A role for brown adipose tissue in diet-induced thermogenesis. *Obesity Research*.

[52] De Macedo I. C., De Freitas J. S., Da Silva Torres I. L. (2016). The influence of palatable diets in reward system activation: A mini review. *Advances in Pharmacological Sciences*.

[53] Bangalore S., Fayyad R., Laskey R., DeMicco D. A., Messerli F. H., Waters D. D. (2017). Body-Weight Fluctuations and Outcomes in Coronary Disease. *New England Journal of Medicine*.

[54] Lissner L., Odell P. M., D'Agostino R. B. (1991). Variability of body weight and health outcomes in the Framingham population. *The New England Journal of Medicine*.

[55] Lissner L., Andres R., Muller D. C., Shimokata H. (1990). Body weight variability in men: Metabolic rate, health and longevity. *International Journal of Obesity*.

[56] Taylor C. B., Jatulis D. E., Fortmann S. P., Kraemer H. C. (1995). Weight variability effects: A prospective analysis from the Stanford five-city project. *American Journal of Epidemiology*.

[57] Knowler W. C., Fowler S. E., Hamman R. F., Christophi C. A., Hoffman H. J., Brenneman A. T. (2009). 10-year follow-up of diabetes incidence and weight loss in the diabetes prevention program outcomes study. *The Lancet*.

